# The Metabolomics Changes in Luria–Bertani Broth Medium under Different Sterilization Methods and Their Effects on *Bacillus* Growth

**DOI:** 10.3390/metabo13080958

**Published:** 2023-08-18

**Authors:** Haifeng Wang, Juan Guo, Xing Chen, Hongxuan He

**Affiliations:** 1 School of Environmental Engineering, Yellow River Conservancy Technical Institute, Kaifeng Key Laboratory of Food Composition and Quality Assessment, Kaifeng 475004, China; 2 National Research Center for Wildlife-Borne Diseases, Key Laboratory of Animal Ecology and Conservation Biology, Institute of Zoology, Chinese Academy of Sciences, Beijing 100101, China

**Keywords:** Luria–Bertani broth medium, 16s rRNA gene sequencing, differential metabolites, microbial growth

## Abstract

Luria–Bertani broth (LB) culture medium is a commonly used bacterial culture medium in the laboratory. The nutrient composition, concentration, and culture conditions of LB medium can influence the growth of microbial strains. The purpose of this article is to demonstrate the impact of LB liquid culture medium on microbial growth under different sterilization conditions. In this study, LB medium with four different treatments was used, as follows: A, LB medium without treatments; B, LB medium with filtration; C, LB medium with autoclaving; and D, LB medium with autoclaving and cultured for 12 h. Subsequently, the protein levels and antioxidant capacity of the medium with different treatments were measured, and the effects of the different LB medium treatments on the growth of microorganisms and metabolites were determined via 16s rRNA gene sequencing and metabolomics analysis, respectively. *Firmicutes* and *Lactobacillus* were the dominant microorganisms, which were enriched in fermentation and chemoheterotrophy. The protein levels and antioxidant capacity of the LB medium with different treatments were different, and with the increasing concentration of medium, the protein levels were gradually increased, while the antioxidant capacity was decreased firstly and then increased. The growth trend of *Bacillus subtilis*, *Bacillus paralicheniformis*, *Micrococcus luteus*, and *Alternaria alternata* in the medium with different treatments was similar. Additionally, 220 and 114 differential metabolites were found between B and C medium, and between C and D medium, which were significantly enriched in the “Hedgehog signaling pathway”, “biosynthesis of plant secondary metabolites”, “ABC transporters”, “arginine and proline metabolism”, and “linoleic acid metabolism”. LB medium may be a good energy source for *Lactobacillus* growth with unsterilized medium, and LB medium filtered with a 0.22 μm filter membrane may be used for bacterial culture better than culture medium after high-pressure sterilization. LB medium still has the ability for antioxidation and to keep bacteria growth whether or not autoclaved, indicating that there are some substances that can resist a high temperature and pressure and still maintain their functions.

## 1. Introduction

Luria–Bertani (LB) broth medium is a complex medium known to promote the structural evolution of subpopulations, mainly containing tryptone, yeast extract and NaCl [1]. LB broth medium can be divided into liquid medium and agar medium. It is not only generally used in biochemical molecular experiments to pre-culture strains, so that strains can be multiplied to meet the requirements of use, but also used to culture genetically engineered recipient bacteria (*Escherichia coli*) [2]. Furthermore, the effects of many bacterial phenotypes can be examined using LB broth medium. A previous study has shown that the growth rate of Iranian Lizard *Leishmania* in RPMI1640 medium and LB broth was similar, and the protein expression in LB medium was higher than that in the other three medium [3]. This indicated that LB broth medium could be a suitable medium for Iranian Lizard *Leishmania* culture and recombinant protein production compared with the RPMI1640 medium, brain heart infusion medium and M199 medium [3]. Another study found that the good growth without senescence of *Podospora anserina* was observed only on LB medium, which prolonged the health of the mycelium, while liquid cultures without senescence were observed only in LB broth medium [4]. Taken together, in life science experiments, LB broth medium is the preferred bacterial growth solution for almost all microbes, including bacteria, fungi, and parasites.

It has been reported that the dilution of LB medium is effective for cultivating bacteria that are unable to culture in undiluted LB medium [5]. Yamamoto et al. [6] showed that the dilution of LB medium could significantly affect the number of colonies, while the bacterial community in 10% LB had more alpha diversity than that in LB. These results indicated that the concentration of LB medium may be critical to support the colony formation of bacteria and to isolate some beneficial bacterial strains [6]. Additionally, LB medium does not contain added glucose or starch, and the yeast extract component is the main energy source. *E. coli* grown in LB medium can lead to gradual changes in the pH and sugar availability, which then affect the cell heterogeneity within microbial communities and the gene expression profiles of microbial populations [7]. A previous study illustrated that the composition of the medium, especially the carbon source, largely determined the pH changes during bacterial growth, and it revealed the main molecular mechanisms behind this phenotype, which may predict the interspecific interactions and adaptability of bacteria in their environment [8]. Another study analyzed the expression profiles of *Dermacoccus abyssi* HZAU 226 cultured in LB medium with or without lysozyme treatment and found 1024 differential expressed genes, including 544 up-regulated and 480 down-regulated genes in response to lysozyme treatment, which were mainly enriched in the pathways of energy metabolism, peptidoglycan biosynthesis, and glutathione biosynthesis and metabolism, which helps to understand the lysozyme-tolerance mechanism of bacteria from a new perspective [9]. All these reports imply that the nutrient composition, concentration, and culture conditions of LB broth medium can influence the growth and development of microbial strains. However, the effects of other different treatments, such as filtration and autoclaving, on the composition of LB medium remain unclear. We usually use high-pressure sterilization to sterilize the culture medium and then conduct experiments. The usual sterilization method is to use high-pressure steam sterilization, and now the sterilization of 0.22 micron filter membrane is gradually increasing. However, due to traditional or customary reasons, high-pressure steam sterilization is still the main sterilization method. Here, it is thought that a high temperature and pressure are more likely to inactivate or destroy chemicals. Therefore, we are wondering if there is a difference in the effect of high-pressure steam sterilization medium and filtered by 0.22 micron filter membrane sterilization medium on bacterial growth under laboratory conditions.

In this study, LB medium with four different treatments was used, as follows: A, the LB medium without any treatments; B, the LB medium filtered with a 0.22 μm filter membrane; C, the LB medium with autoclaving; and D, the LB medium with autoclaving and then cultured for 12 h. After that, the relevant protein levels and antioxidant capacity of the medium with different treatments were measured, while the effects of LB medium with different treatments on the growth of microorganisms and metabolites were determined via 16s rRNA gene sequencing and metabolomics analysis, respectively. This study can provide new insights and views for the enrichment culture of microorganisms.

## 2. Materials and Methods

### 2.1. Treatment of LB Broth Medium

The LB broth medium contained 10 g/L peptone, 3 g/L beef extract powder, and 5 g/L NaCl, and it was purchased from Hepebio Co., Ltd. (Qingdao, China). The configuration method of the LB medium was as follows: 1.8 g LB broth medium dissolved in 100 mL ultra-pure water.

The LB broth medium was divided into four groups: A, B, C, and D groups. In the A group, the LB broth medium was prepared without any treatments. In the B group, the prepared medium was filtered with a 0.22 μm filter membrane. The medium in the C group was autoclaved at 121 °C for 20 min using an autoclave (GI80PS, ZEALWAY, Xiamen, China). The medium in the D group was firstly autoclaved and then maintained at 37 °C for 12 h on a shaking table with 300 rpm/min.

In addition, for the 16s rRNA sequencing, the prepared unsterilized LB broth medium (A group) was divided into two groups: A1 (A-3, A-5, A-6 and A-7) and A2 (A-I, A-II, A-III, A-IV, A-V, and A-VI) groups. In the A1 group, 5 mL LB broth medium was added to a 50 mL centrifuge tube, which then was maintained on a shaking table with 300 rpm/min at 37 °C for 24 h. In the A2 group, 1 mL LB broth medium was added to each well of a 48-well plate and then cultured on a shaking table with 600 rpm/min at 37 °C for 24 h. After that, the medium solution with different treatments was collected and centrifuged at 12,000 rpm for 5 min at 4 °C. After removing the supernatant, the sediments were placed in liquid nitrogen for 30 min and then immediately stored at −80 ℃. Finally, the sediment samples were sent to YINGZI GENE (Wuhan, China) for 16s rRNA gene sequencing.

### 2.2. 16S rRNA Gene Sequencing and Analysis

The collected samples in the A1 and A2 groups were used to isolate the total genomic DNA using a TIANamp Bacteria DNA Kit (TIANGEN, Beijing, China) and then qualified with 1% agarose gel electrophoresis. Following, the DNA samples were used to amplify the V3–V4 region of the 16S rRNA gene with primers (F: ACTCCTACGGGAGGCAGCA; R: GGACTACHVGGGTWTCTAAT). The PCR products were purified using an Agencourt AMPure XP nucleic acid purification kit, and they were submitted for 16S rRNA gene sequencing using the Illumina NovaSeq platform at YINGZI GENE.

After the raw data were dismounted, the results were stored in Fastq format and then Trimmonmatic (version 0.36), Pear (version 0.9.6), Flash (version 1.20), and Vsearch (version 2.7.1) software were employed for quality control, and the high-quality data were obtained [10]. After that, the high-quality data were used to generate Operational Taxonomic Units (OTUs) using clustering (or noise reduction) with QIIME2 software (version 1.8.0). All the sequences can be divided into OTUs according to different similarity levels, and bioinformatics statistical analysis is usually performed for OTUs at the 97% similarity level [11]. In order to obtain the species classification information corresponding to each OTU, the RDP Classifier algorithm was used to compare and analyze the representative sequences of OTUs and annotate the species information at the phylum, class, order, family, genus, and species levels [12]. Then, the QIIME2 software (version v.1.8.0, http://qiime.org/scripts/alpha_rarefaction.html, accessed on 7 April 2023) was used to analyze the α-diversity indexes, and PCA (Principal Component Analysis) statistical analysis in the R language was employed for the β-diversity analysis [13]. Finally, the identified bacteria were used for functional analysis using FAPROTAX software (version 1.2.5), and the functional pathways were clustered using Heatmap [14].

### 2.3. Measurement of the Protein Concentrations and Antioxidant Capacity

Based on the method of Bradford [15], the protein concentrations of the samples in the A, B, C and D groups were measured using a Quick Start^TM^ Bradford Protein Assay (Bio-Rad, Hercules, CA, USA). Furthermore, a Total Antioxidant Capacity Assay Kit with the ABTS method (Beyotime Biotechnology, Shanghai, China) was employed to determine the total antioxidant capacity of the samples in the different groups according to the manufacturer’s instructions.

### 2.4. Bacterial Inoculation Experiment

Because the medium in the A group could grow bacteria without being inoculated with bacteria, it was not used for the inoculation experiments with different bacteria. Therefore, the medium in the B, C, and D groups was used to inoculate different bacteria at different concentrations, including *Bacillus subtilis*, *Bacillus paralicheniformis*, *Micrococcus luteus*, and *Alternaria alternata* [16]. All the bacteria were inoculated in the LB medium and cultured to the exponential phase. Then, the plate colony counting experiment was performed, and the initial bacterial concentration of each bacterium was adjusted to 1 × 10^8^/mL for the subsequent growth experiments with different volume inoculation (1 μL, 10 μL, 20 μL, 40 μL, 60 μL, 80 μL, and 100 μL) in the different medium (B, C, and D medium). After being cultured for 70 h, the optical density in the different groups was measured at 600 nm using a microbial high-throughput growth detector (MicroScreen-HT, JIELING, Tianjin, China) every hour.

### 2.5. Metabolite Extraction and Metabolomics Analysis

The samples in the B, C and D groups were used for the metabolomic analysis. The medium samples (100 μL) were added to 400 μL methyl alcohol. After vortexing for 1 min, the samples were centrifuged at 12,000 rpm for 10 min at 4 °C, and the supernatant was transferred to a new tube. After concentration and drying, the powder samples were redissolved in 2-chloro-l-phenylalanine (4 ppm) solution prepared with 80% methanol water (150 µL) and then filtered through a 0.22 μm membrane. The filtered samples were submitted for the determination of liquid chromatography–mass spectrometry (LC–MS).

A Vanquish UHPLC system (Thermo Fisher Scientific, Waltham, MA, USA) equipped with an ACQUITY UPLC^®^ HSS T3 column (1.8 µm, 2.1 × 150 mm; Waters, Milford, MA, USA) and a mass spectrometer (Q Exactive Focus; Thermo Fisher Scientific) was used for the LC–MS detection. The flow rate was 0.25 mL/min, the temperature of column was maintained at 40 °C, and the injection volume was 2 μL. The mobile phases for the positive were 0.1% formic acid in acetonitrile (*v*/*v*, B2) and 0.1% formic acid in water (A2), and for the negative, they were 5 mM ammonium formic water (A3) and acetonitrile (B3). The spray voltage of the MS for the positive and negative modes was, respectively, 3.5 kV and 2.5 kV; the capillary temperature was 325 °C; and sheath gas and auxiliary gas were set at 30 and 10 arbitrary units. Full scanning was performed at a resolution of 70,000 and a scanning range of 100–1000 *m*/*z*.

The MSConvert tool in the Proteowizard software (v3.0.8789) was used to transform the original data from LC–MS to an mzXML format, and the CXMS package of R was used for the peaks detection, peaks filtration, and peaks alignment with the parameters of bw = 2, ppm = 15, peakwidth = c (5, 30), mzwid = 0.015, mzdiff = 0.01 and method = “centWave” [17]. Then, the metabolites were identified according to the public databases HMDB, massbank, LipidMaps, mzcloud, and KEGG, and the parameter was set as ppm < 30 ppm. The Ropls package in R [18] was used for all the multivariate data analyses and modelling, and significantly differential metabolites were screened based on the thresholds of VIP > 1 and *p* < 0.05. Finally, a Kyoto Encyclopedia of Genes and Genomes (KEGG) pathway enrichment analysis [19] was carried out on these screened differential metabolites.

### 2.6. Statistical Analysis

Each experiment was repeated three times, and the data are reported as the mean ± standard deviation. GraphPad Prism 5 (GraphPad Software, San Diego, CA, USA) was used for the statistical analyses and to draw the figures. The differences between two groups of data were examined with Student’s *t* test, and for the differences among more than two groups, a one-way ANOVA followed by Tukey’s post hoc test were conducted. A *p* < 0.05 indicated a statistical difference.

## 3. Results

### 3.1. Microbiome Changes in the LB Medium with Different Treatments

**Figure 1:**
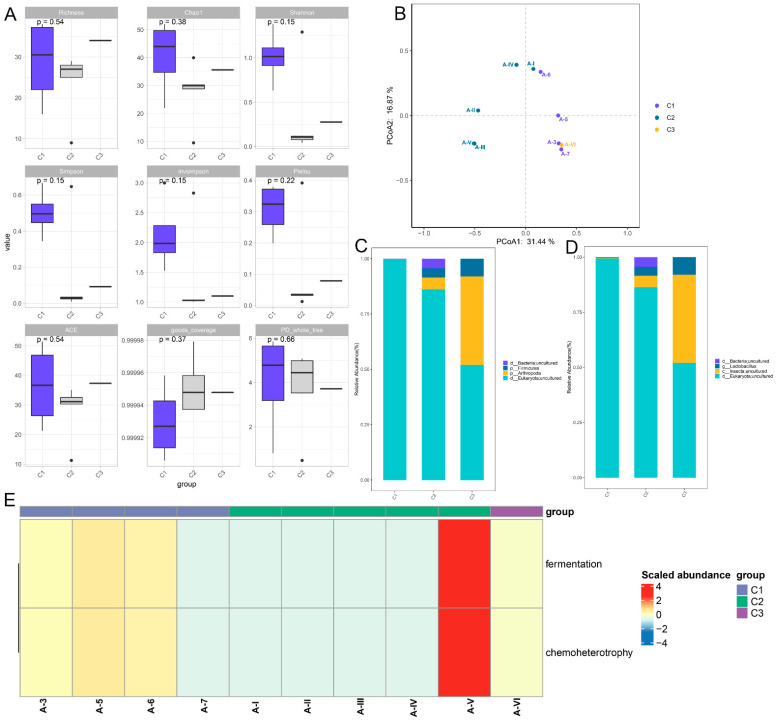
Microbiome changes in the LB medium with different treatments. (**A**) The alpha diversity analyses based on the indexes of Richness, Chao1, Shannon, Simpson, invsimpson, Pielou, ACE, Good coverage, and PD-whole tree. (**B**) Principal coordinates analysis of all the samples. (**C**) The bacterial communities at the phylum level. (**D**) The bacterial communities at the genus level. (**E**) Functional analysis of the identified bacterial communities. C1: the samples of A-3, A-5, A-6 and A-7 in the A1 group. C2: the samples of A-I, A-II, A-III, A-IV and A-V in the A2 group. C3: the A-VI sample in the A2 group.

Through 16S rRNA gene sequencing, there were no significant differences in the indexes of Richness, Chao1, Shannon, Simpson, invsimpson, Pielou, ACE, Good coverage, and PD-whole tree between the different groups (Figure 1A). Principal coordinates analysis (PCoA) showed that the samples of A-3, A-5, A-6 and A-7 in the A1 group (C1) could be separated from the samples of A-I, A-II, A-III, A-IV and A-V in the A2 group (C2); while the A-VI sample in the A2 group (C3) was clustered in the C1 (Figure 1B). Then, the microbiota were analyzed at the phylum and genus levels. From the phylum level, four dominant phyla were observed, including Bacteria-uncultured and Firmicutes (Figure 1C). Furthermore, from the genus level, Bacteria-uncultured, *Lactobacillus*, and Insecta-uncultured were found; the *Lactobacillus* abundance was higher in the C2 samples and A-VI samples compared with the C1 group (Figure 1D). Finally, the identified microbiota were used for functional analysis, and it was found that fermentation and chemoheterotrophy were significantly enriched (Figure 1E). When other species have also been found in microbial polymorphism experiments, they are likely to be the result of a mismatch or high similarity in gene sequences.

### 3.2. Effects of Different Treatments on the Protein Level and Antioxidant Capacity of LB Medium

**Figure 2:**
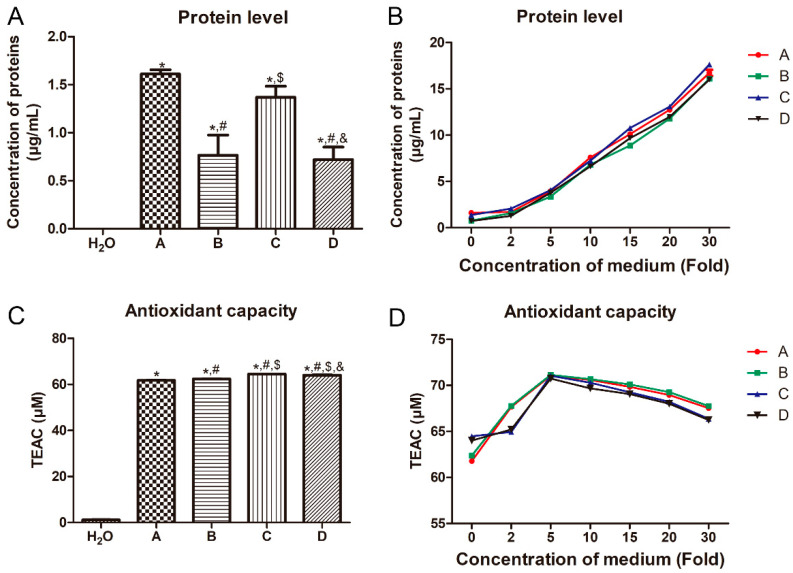
Effects of different treatments on the protein level and antioxidant capacity of LB medium. (**A**) The protein levels in the medium with different treatments at a concentration of 1×. (**B**) The protein levels in the medium with different treatments at different concentrations. (**C**) The antioxidant capacity in the medium with different treatments at a concentration of 1×. (**D**) The antioxidant capacity in the medium with different treatments at different concentrations. TEAC: Trolox-equivalent antioxidant capacity. *: *p* < 0.05, compared with the H_2_O; ^#^: *p* < 0.05, compared with the A medium; ^$^: *p* < 0.05, compared with the B medium; ^&^: *p* < 0.05, compared with the C medium.

The effects of the different treatments on the protein levels and antioxidant capacity of the LB medium were determined. Compared with the H_2_O, the protein levels in the A, B, C, and D medium were all significantly increased (*p* < 0.05, Figure 2A). No significant difference in the protein level was found between the B and D medium, as well as the A and C medium (*p* > 0.05, Figure 2A). Compared with the C medium, the protein level in the D medium was significantly decreased (*p* < 0.05, Figure 2A). This may be due to the 12 h of empty shaking. Then, we measured the protein levels in the medium with different treatments at different concentrations. It was found that with the increasing concentration of the medium, the protein level of the medium was gradually elevated (Figure 2B).

In addition, the antioxidant capacity of the medium with different treatments was evidently higher than that in the H_2_O (*p* < 0.05, Figure 2C). Compared with the A medium, the antioxidant capacity of the B, C and D groups was also markedly increased (*p* < 0.05, Figure 2C). Compared to the C medium, the antioxidant capacity of the D medium was significantly reduced (*p* < 0.05, Figure 2C). With the increased concentration of the medium, the antioxidant capacity of the medium with different treatments was decreased firstly and then increased, while the antioxidant capacity of the medium with different treatments was lowest at the medium concentration of 5× (Figure 2D).

### 3.3. Growth Ability of Different Bacteria on the LB Broth Medium

**Figure 3:**
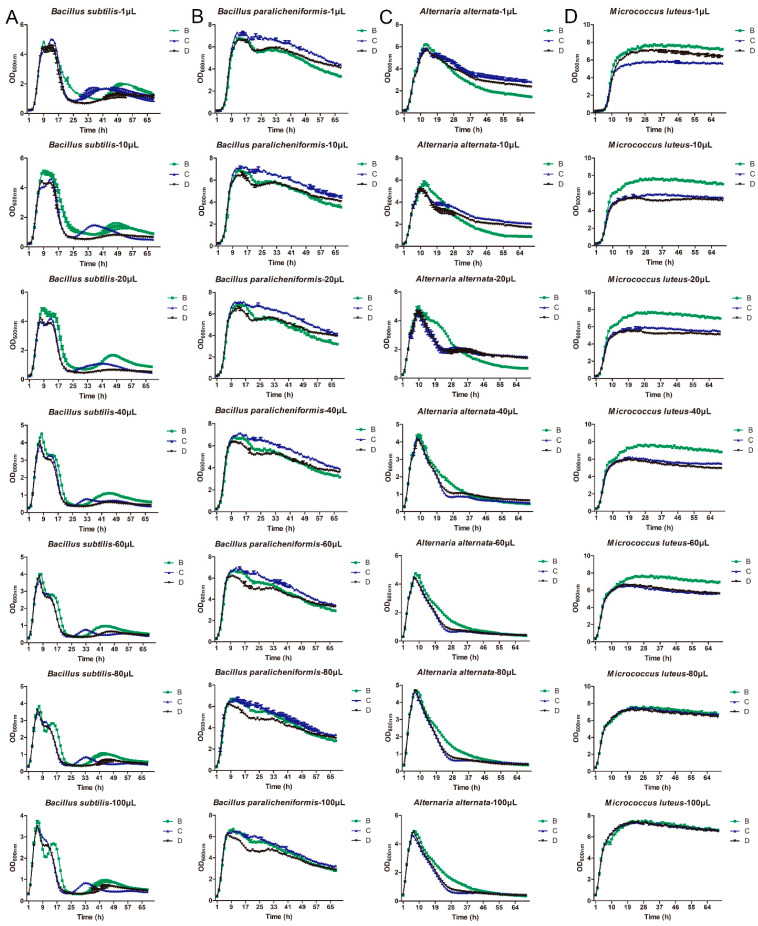
Growth ability of different bacteria on the LB broth medium with different treatments. The growth of *Bacillus subtilis* (**A**), *Bacillus paralicheniformis* (**B**), *Alternaria alternata* (**C**), and *Micrococcus luteus* (**D**) at different concentrations in the LB medium with different treatments after being cultured for different times. B medium: the LB medium filtered with a 0.22 μm filter membrane; C medium: the LB medium autoclaved at 121 °C for 20 min; D medium: the LB medium firstly autoclaved, and then cultured for 12 h.

*B. subtilis*, *B. paralicheniformis*, *M. luteus*, and *A. alternata* at different concentrations were inoculated in the medium with different treatments and cultured for different times. For *B. subtilis*, it had a better growth advantage in B medium, as well as with the increase in the culture time, the OD value of its growth was first increased and then decreased, and then it tended to be stable (Figure 3A). The peak point of *Bacillus subtilis* at 1, 10, 20, 40, 60, 80, and 100 μL was cultured at 9 h, 9 h, 8 h, 8 h, 7 h, 7 h, and 6 h, respectively (Figure 3A). For *B. paralicheniformis*, it had a better growth advantage in C medium, and the OD value of its growth was first increased and then decreased with the increasing culture time, while the peak point of *B. paralicheniformis* at 1, 10, 20, 40, 60, 80, and 100 μL was cultured at 13 h, 13 h, 14 h, 11 h, 11 h, 10 h, and 10 h, respectively (Figure 3B). The trend of the OD value of *A. alternata* had the highest number of bacteria in B medium, which was similar to that of *Bacillus subtilis*, and the peak point of *Alternaria alternata* at 1, 10, 20, 40, 60, 80, and 100 μL was cultured at 13 h, 12 h, 10 h, 9 h, 8 h, 8 h, and 7 h, respectively (Figure 3C). Additionally, for *M. luteus*, with the increasing culture time, the OD value of its growth was first increased and then tended to be stable after cultured for about 19 h at different concentrations of *M. luteus* (Figure 3D). When the concentration of *M. luteus* was low, it had the best growth advantage in B medium, followed by D medium and C medium. However, with the increase in the concentration of *M. luteus*, the growth advantages of the B medium and C medium were gradually approaching.

### 3.4. Differential Metabolites between the Medium in the B and C Groups, and Functional Analysis

**Figure 4:**
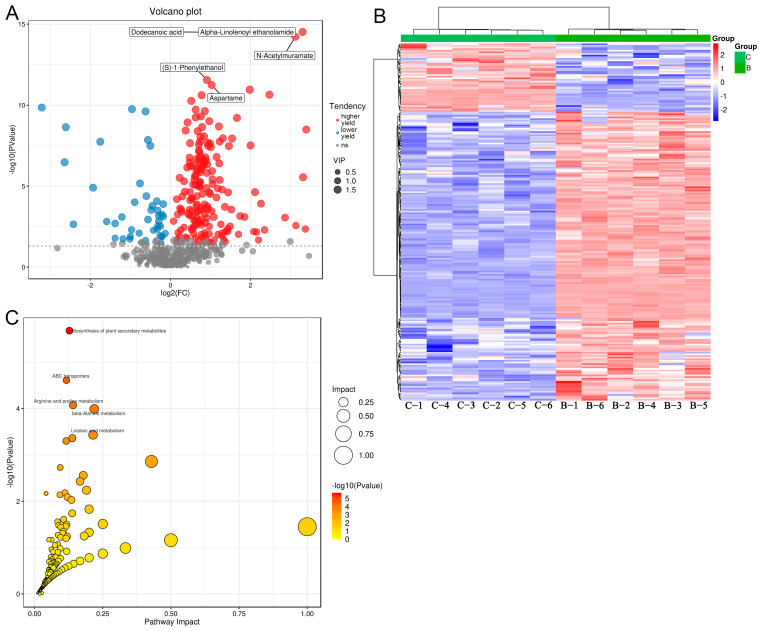
Identification of differential metabolites between the medium in the B and C groups, and functional analysis. (**A**) The volcano plot of the differential metabolites between the B and C medium. (**B**) The clustering heatmap of the identified differential metabolites between the B and C medium. (**C**) The KEGG pathways map of the identified differential metabolites between the B and C medium.

Furthermore, to investigate the differential metabolites among the B, C, and D medium, metabolomics was conducted. Based on VIP > 1 and *p* < 0.05, 220 differential metabolites were screened between the B and C medium, including 178 metabolites with higher yields and 42 metabolites with lower yield in the B medium, such as dodecanoic acid, alpha-linolenoyl ethanolamide, N-acetylmuramate, (S)-1-phenylethanol, and aspartame (Figure 4A). The clustering heatmap of these 220 differential metabolites is displayed in Figure 4B, which shows that the identified differential metabolites could significantly distinguish the B medium from the C medium.

The KEGG is a database of systematic analyses of gene function and genomic information. The identified differential metabolites were used for KEGG pathway enrichment analysis. Based on the results in Figure 4C, we found that the differential metabolites were significantly enriched in the pathways of “Hedgehog signaling pathway”, “biosynthesis of plant secondary metabolites”, “ABC transporters”, “arginine and proline metabolism”, “beta-alanine metabolism”, and “linoleic acid metabolism”.

### 3.5. Differential Metabolites between the Medium in the C and D Groups, and Functional Analysis

**Figure 5:**
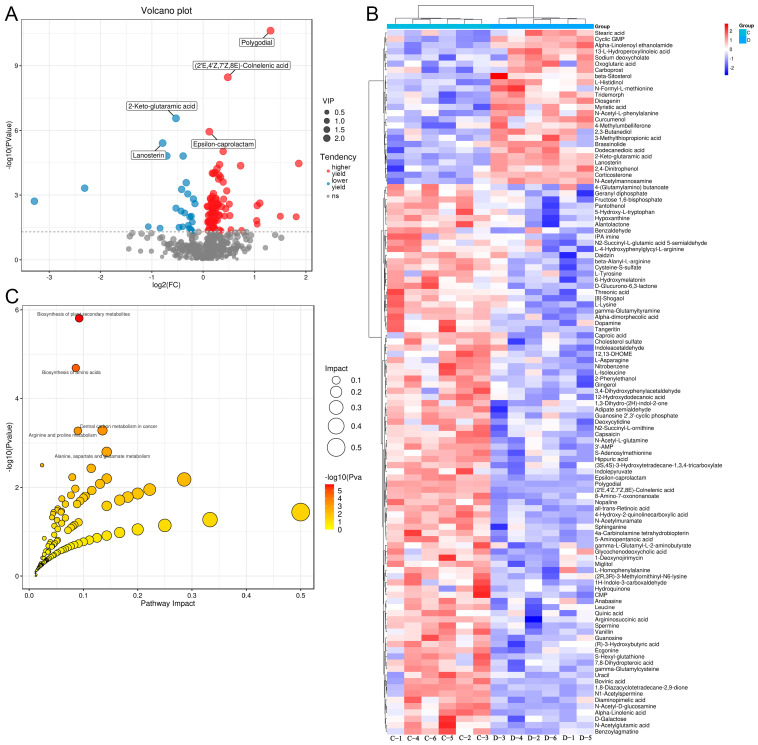
Identification of differential metabolites between the medium in the C and D groups, and functional analysis. (**A**) The volcano plot of the differential metabolites between the C and D medium. (**B**) The clustering heatmap of the identified differential metabolites between the C and D medium. (**C**) The KEGG pathways map of the identified differential metabolites between the C and D medium.

According to VIP > 1 and *p* < 0.05, 114 differential metabolites were screened between the C and D medium, including 89 metabolites with higher yield (polygodial, (2′E,4′Z,7′Z,8E)-Colnelenic acid, and epsilon-caprolactam) and 25 metabolites with lower yield (2-keto-glutaramic acid, and lanosterin) in the D medium (Figure 5A). The clustering heatmap of the 114 differential metabolites displayed that the C and D medium could be well differentiated by the identified differential metabolites (Figure 5B).

After that, the identified differential metabolites were subjected to functional analysis, and based on *p* < 0.05, 37 KEGG pathways were significantly enriched, including “biosynthesis of plant secondary metabolites”, “biosynthesis of amino acids”, “central carbon metabolism in cancer”, “arginine and proline metabolism”, “alanine, aspartate and glutamate metabolism”, “linoleic acid metabolism”, and “intestinal immune network for IgA production” (Figure 5C).

## 4. Discussion

LB medium is one of the most powerful media for cultivating bacteria, although the effects of LB broth sterilization medium on microbial growth and metabolites under different conditions remain unclear. Due to the fact that high-pressure steam sterilization usually uses electric heating and steam to eliminate the cooling process, and the total process takes a certain amount of time, we need to find a method that can conduct experiments without affecting the experimental results in situations such as power interruption, malfunction of the high-pressure sterilizer, missed detection period of the high-pressure sterilizer uncertainty the effectiveness of the instrument, and time constraints, etc. Our experiments demonstrated that LB medium filtered with a 0.22 μm filter membrane may be a more effective and time-saving method in general laboratory scientific operations. Our study was the first to investigate the roles of different treatments in the bacterial growth and metabolite changes in LB medium. It was found that *Firmicutes* was the dominant phyla and *Lactobacillus* was the preponderant genus, which were enriched in the pathways of fermentation and chemoheterotrophy. The protein levels and antioxidant capacities of the LB medium with different treatments were different, as with the increasing concentration of the medium, the protein level was gradually increased, while the antioxidant capacity was increased firstly and then decreased. The growth trend of *B. subtilis*, *B. paralicheniformis*, *M. luteus*, and *A. alternata* in the B, C and D medium was similar, which indicated that LB medium filtered with a 0.22 μm filter membrane could also be used for bacterial culture. Additionally, 220 differential metabolites between B and C medium, and 114 differential metabolites between C and D medium, were found, which were significantly enriched in the “Hedgehog signaling pathway”, “biosynthesis of plant secondary metabolites”, “ABC transporters”, “arginine and proline metabolism”, “intestinal immune network for IgA production”, and “linoleic acid metabolism”.

Through 16s rRNA gene sequencing, *Firmicutes*, and *Lactobacillus* were detected in the unsterilized LB broth culture medium, and they were enriched in the pathways of fermentation and chemoheterotrophy. *Firmicutes* is one of the most abundant groups of prokaryotes in the human and animal microbiota, including genera of outstanding relevance in biomedicine, healthcare, and industry [20]. Gavande et al. [21] reported that *Firmicutes* could play a key role in the deconstruction of lignocellulose and in the degradation of hemicellulose. *Lactobacillus* are a variety of gas-tolerant and glycololytic microorganisms in *Firmicutes*, which mainly use fermentation to save energy [22]. Extracellular electron transfer can increase the fermentation of *Lactobacillus* through hybridization metabolism. Furthermore, *Lactobacillus* is the genus of bacteria that contains the most characteristic probiotics, and the bacteria can often utilize a variety of carbohydrates. This feature is essential to their survival in highly competitive environments, such as the gastrointestinal tract of animals [23]. A previous study showed that the alternative nitrogen sources X-Seed Nucleo Max, X-Seed KAT, and X-Seed Carbo Max could significantly support the growth of *Lactobacillus* [24]. Chemoheterotrophy is also an ecological function of microorganisms. Taken together, we can speculate that LB broth medium without sterilization may be a good energy source for the growth of *Lactobacillus*, which may play important roles through fermentation and chemoheterotrophy.

Protein can provide a source of nitrogen for bacterial growth and reproduction. Oxidative stress can occur in all strains during culture and produce oxidative intermediates, which is more obvious in serum-free medium systems due to the lack of antioxidant substances [25]. With the increase in the reactive oxygen species concentration, superoxide free radicals and hydrogen peroxide will be produced, and these molecules will damage lipids, proteins and DNA, and also cause cell damage [26]. Therefore, when designing the medium, on the one hand, antioxidant substances need to be used to enable cells to have the ability to resist the oxidative environment, and on the other hand, the probability of the oxidation of unstable components in the medium is reduced [27]. Combined with our results, it can be inferred that different treatments could affect the protein level and antioxidant capacity of LB medium, although they may not significantly influence the growth of bacteria. This indicated that whether autoclaved or not, there may be some substances in LB medium that can resist the ability of high temperature and pressure. Additionally, the protein level and antioxidant capacity in the autoclaved medium (C medium) were both higher than those in the 12 h incubated medium (D medium). We speculated that this may be due to the effects of 12 h of empty shaking on the changes in the related substances in the LB medium. However, the specific reasons need to be further explored.

In addition, we further investigated the differential metabolites and related enriched pathways among the medium with different treatments, and it was found there were 220 and 114 differential metabolites between B and C medium, as well as between C and D medium, respectively. The Hedgehog signaling pathway is an evolutionarily conserved pathway for signaling from the cell membrane to the nucleus, and it plays an important role in the normal embryonic development of invertebrates and vertebrates [28]. Fu et al. [29] demonstrated that the alpha-hemolysin produced by *Escherichia coli* in meningitis could exacerbate blood–brain barrier disruption by targeting the TGFβ1-triggered Hedgehog signaling pathway. Plant secondary metabolites are not only a range of useful natural products but also an important part of plant defense systems against pathogenic attacks and environmental stress [30]. A previous study revealed that 19 secondary metabolites of *B. amyloliquefaciens*, including iturins, fengycins, and surfactin, had inhibitory effects on the necrotrophic fungus *Botrytis cinerea* and could be used as an antifungal agent [31]. ABC transporters are the major transporters that pair energy stored in adenosine triphosphate (ATP) to the movement of molecules across the membranes, and they are associated with multiple drug resistance in bacteria and eukaryotic cells [32]. Amino acids are the molecular basis of protein synthesis by cells, and arginine and proline are two important amino acid. Raza et al. [33] illustrated that the gut microbiota could play crucial roles in promoting host resistance to the low temperature stress of *Bactrocera dorsalis* by stimulating their arginine and proline metabolic pathways. Linoleic acid can be converted into conjugated linoleic acid by several microorganisms, including *Bifidobacterium* and *Lactobacillus*, possibly as a detoxification mechanism to avoid the growth-inhibiting effects of linoleic acid [34]. A study by Peng et al. [35] showed that linoleic acid was the key metabolite of *Lactobacillus*, and *Lactobacillus* with excess linoleic acid could limit the growth, survival, and virulence of *Salmonella typhimurium* and enterohemorrhagic *E. coli*. Additionally, the intestinal immune network for IgA production has been found to be dysregulated in the lung metastasis of colorectal cancer [36], and the role in bacterial growth has not been reported. The analysis of the nutritional components of the LB culture medium under different treatment conditions through metabolomics experiments provided our further understanding of the impact of the culture medium components on microbial growth in microscale. Metabolomics experiments showed that we found changes in 220 and 114 chemical components under the culture medium with different treatment conditions. Through the KEGG analysis of the differential chemical components, it was found that the culture medium under different treatment conditions had both the same functional pathways, such as “biosynthesis of plant secondary metabolites”, “arginine and proline metabolism”, and “linoleic acid metabolism”, and different functional pathways, such as “Hedgehog signaling pathway”, “ABC transporters”, “aspartate and glutamate metabolism”, and “linoleic acid metabolism”. These findings indicate that differences in treatment conditions do indeed affect the chemical composition, although there is no significant change in the growth of *Bacillus* under laboratory conditions. These reports, together with our results, mean we can assume that different treatments may affect the metabolites of LB broth medium and may regulate the pathways of the Hedgehog signaling pathway, biosynthesis of plant secondary metabolites, ABC transporters, arginine and proline metabolism, intestinal immune network for IgA production, and linoleic acid metabolism. However, the specific roles and mechanisms of the differential metabolites and pathways in the LB medium with different treatments need to be further explored.

## 5. Conclusions

LB is a very ordinary culture medium, although we are not very clear about its substance composition. Our experiment is the first to analyze the substance and function of LB medium under different conditions. The purpose of this article is to demonstrate the impact of LB liquid culture medium on microbial growth under different sterilization conditions. Changes in the treatment conditions of the culture medium can lead to changes in the chemical composition. LB broth medium may be a good energy source for the growth of *Lactobacillus* when not sterilized. The protein concentration increases with the concentration of the culture medium, although the antioxidant capacity is not the same as the protein concentration. An important finding is that the antioxidant capacity of LB does not differ significantly under high pressure or not. The growth experiments of *Bacillus subtilis* and *Bacillus licheniformis* found that the effect of different treatment conditions on *Bacillus* growth is not very significant, which means that both membrane sterilization and high-pressure sterilization can be applied under laboratory conditions. However, further research is still needed if applied to industrialization. Our experiments demonstrated LB medium filtered with a 0.22 μm filter membrane may be a more effective and time-saving method, although the difference from high-pressure sterilization is not very significant in general laboratory scientific operations. Our study was just a preliminary study, and the effects of medium with different treatments on the growth of *E. coli* or other bacteria require to be further investigated, while whether the filtered LB medium is suitable for all other bacteria also needs to be studied. The findings of our experiments provide us with a new understanding of the nutrient tolerance, antioxidant capacity, and adaptability of LB medium.

## Data Availability

Data is presented within the article.
